# Top or Middle? Up or Down? Toward a Standard Lexicon for Protein Top-Down and Allied Mass Spectrometry Approaches

**DOI:** 10.1007/s13361-019-02201-x

**Published:** 2019-05-09

**Authors:** Frederik Lermyte, Yury O. Tsybin, Peter B. O’Connor, Joseph A. Loo

**Affiliations:** 1 School of Engineering, University of Warwick, Coventry, CV4 7AL UK; 2 Department of Chemistry, University of Warwick, Coventry, CV4 7AL UK; 3 Spectroswiss, EPFL Innovation Park, 1015 Lausanne, Switzerland; 4 Department of Chemistry and Biochemistry, Department of Biological Chemistry, David Geffen School of Medicine, and UCLA/DOE Institute of Genomics and Proteomics, University of California, Los Angeles, CA USA

**Keywords:** Top-down mass spectrometry, Top-down proteomics, Native mass spectrometry, Proteoform

## Abstract

In recent years, there has been increasing interest in top-down mass spectrometry (TDMS) approaches for protein analysis, driven both by technological advancements and efforts such as those by the multinational Consortium for Top-Down Proteomics (CTDP). Today, diverse sample preparation and ionization methods are employed to facilitate TDMS analysis of denatured and native proteins and their complexes. The goals of these studies vary, ranging from protein and proteoform identification, to determination of the binding site of a (non)covalently-bound ligand, and in some cases even with the aim to study the higher order structure of proteins and complexes. Currently, however, no widely accepted terminology exists to precisely and unambiguously distinguish between the different types of TDMS experiments that can be performed. Instead, ad hoc developed terminology is often used, which potentially complicates communication of top-down and allied methods and their results. In this communication, we consider the different types of top-down (or top-down-related) MS experiments that have been performed and reported, and define distinct categories based on the protocol used and type(s) of information that can be obtained. We also consider the different possible conventions for distinguishing between middle- and top-down MS, based on both sample preparation and precursor ion mass. We believe that the proposed framework presented here will prove helpful for researchers to communicate about TDMS and will be an important step toward harmonizing and standardizing this growing field.

**Figure:**
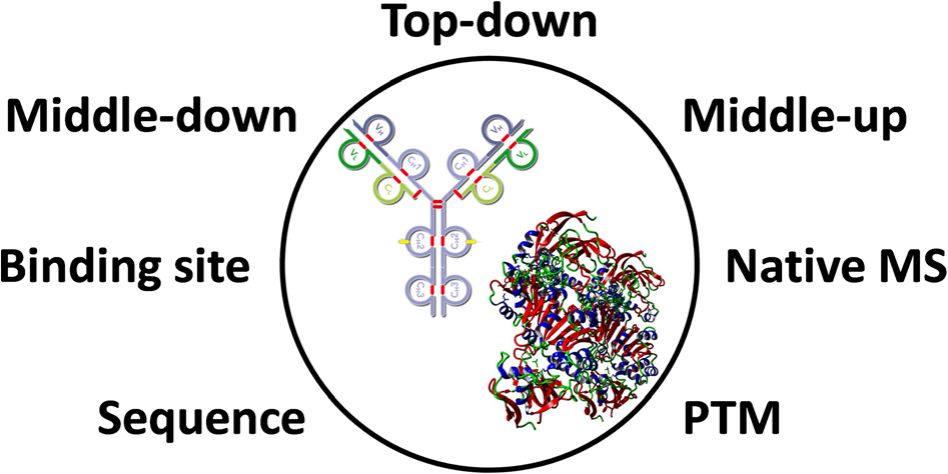
Graphical Abstract

Graphical Abstract

## Introduction

The past decade has witnessed tremendous progress in both top-down (TD) and native protein mass spectrometry (MS) [1]. Largely, this has been driven by technological evolutions, including improvements in ionization techniques, reduced-frequency multipoles for high-*m/z* transmission and isolation, and high-*m/z* detectors, e.g., time-of-flight and extended-mass-range ion cyclotron resonance (ICR) and Orbitrap Fourier transform mass spectrometry (FTMS) [2, 3]. Likewise, the increased availability of sophisticated ion activation techniques (e.g.*,* electron-based dissociation [4] and ultraviolet photodissociation [5]) and high-resolution MS (a requirement for the analysis of complex, crowded product ion spectra) have allowed TDMS and even its large-scale application, top-down proteomics, to become more mainstream, as evidenced by the recent establishment of the multinational Consortium for Top-Down Proteomics (CTDP) [6]. Both native and top-down MS have focused on the analysis of ever-larger and more complex proteins and, in recent years, research has been carried out by some groups on the interface between these two fields. In these studies, gas-phase dissociation of noncovalent protein-protein and protein-ligand complexes has yielded a wealth of information, as recently reviewed [4].

A result or side effect of the increased interest in this relatively new field is the use of occasionally inconsistent, ad hoc developed terminology in the literature. For example, the term “native electron capture dissociation” (native ECD) was originally introduced in 2003 by Breuker and McLafferty to refer to a process presumably initiated by asymmetric charge partitioning during dissociation of the cytochrome c dimer within a heated transfer capillary (i.e., without the introduction of free low-energy electrons) and has recently been used by Kelleher and colleagues to describe essentially the same process occurring in the native ferritin complex [7–9]. However, the term “native ECD” has also been used by others to describe experiments in which a folded protein complex was irradiated with low-energy electrons within an ion cyclotron resonance (ICR) cell [10–12], a process that is obviously fundamentally different from that reported by Breuker and McLafferty.

Here, we make the case for a standardized, unambiguous lexicon to describe the different variants of native and non-native TDMS experiments. We propose a possible “taxonomy” of TDMS approaches, highlighting those types of experiments that are of particular relevance to our own research interests. We are conscious of the fact that others may wish to expand upon our proposed lexicon in time. Our hope is that this work, like the unambiguous definition of the term “proteoform” in 2013 [13] and the recent introduction of the ProForma notation [14], will be an important step toward the full regularization of the top-down field. The focus here is on protein analysis, but of course, as TDMS is extrapolated to other types of large biomolecules, such as nucleic acids, the proposed nomenclature can be applied to these types of molecules.

As most TDMS studies so far have been performed using electrospray ionization (ESI), our discussion will focus on protein ions generated in this way, although some of these experiments can also be performed using other methods, e.g.*,* matrix-assisted laser desorption/ionization (MALDI). For experiments in which controlled backbone cleavage occurs (which we will argue is a requirement to be considered TDMS), different terms are introduced depending on whether the protein is measured from denaturing or non-denaturing solution, and in the latter case, whether higher order structure is retained during backbone cleavage. In what follows, we will strike a balance between introducing a sufficiently precise vocabulary so that most types of TDMS experiments can be referred to unambiguously, while not introducing such a number of new terms that our nomenclature becomes unwieldy. By necessity, and as will be discussed later on, this will leave some degree of ambiguity, particularly in regard to the more “niche” experiments. We emphasize, however, that the lexicon introduced here is meant to be flexible and extendable by others, should the need arise in the future. The terms we introduce or define unambiguously to denote different approaches are, in approximate order of increasing experimental complexity:

- Intact mass measurement
- Native MS
- Complex-up
- Middle-up
- Middle-down
- (Denaturing) top-down (dTD or TD)
- Complex-down
- Native top-down (nTD)

**Figure 1:**
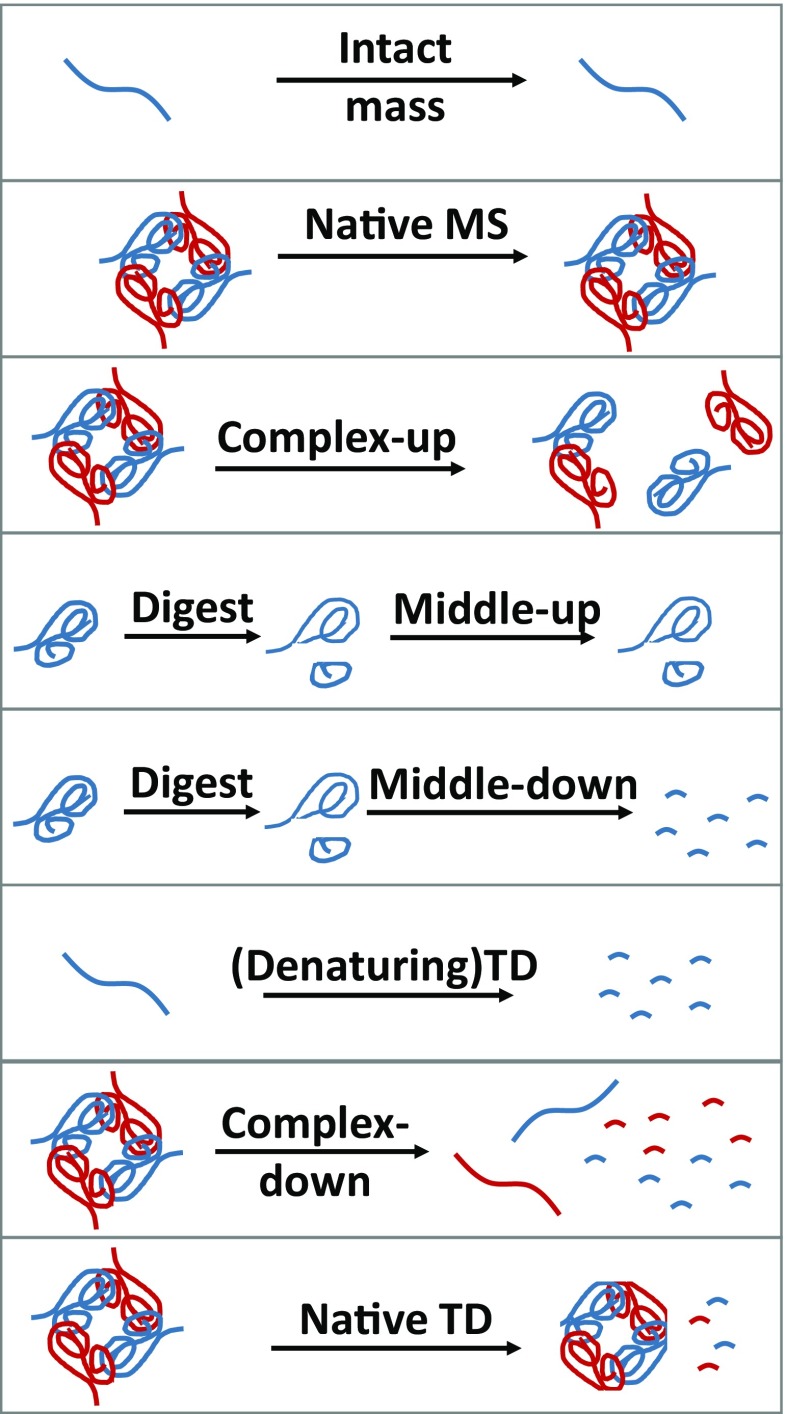
Various (non-)native top-down and allied methods as defined in this work

**Figure 2:**
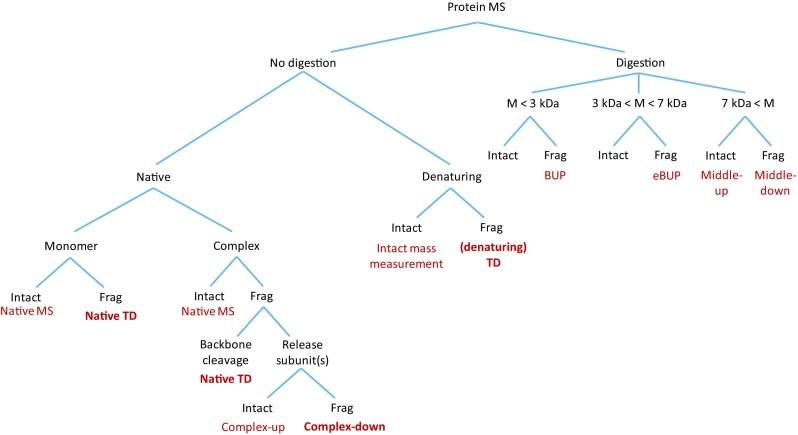
How the methods described in Figure 1 relate to one another, as well as to (extended) bottom-up approaches. The “true” top-down methods are given in bolded text

Only the latter three should be considered “true” top-down methods, as only they inform on primary protein structure (vide infra), although the former types of experiment also provide highly valuable information. A cartoon representation of each of these eight types of experiment is provided in Figure 1, and their relation to one another, as well as to (extended) bottom-up methods, is illustrated in Figure 2. An overview of methods not relying on enzymatic digestion is provided in tabular form in Table 1.

**Table 1:** Overview of the MS Methods Without Enzymatic/Chemical Digestion That We Define in This Manuscript, with the Bottom Three Making Up the “True” Top-Down Methods

	Buffer	Backbone fragmentation	Tertiary structure destroyed	Quaternary structure destroyed
Intact mass	Denaturing	No	N/A	N/A
Native MS	Native	No	No	No
Complex-up	Native	No	Yes/no	Yes*
Denaturing TD	Denaturing	Yes	N/A	N/A
Complex-down	Native	Yes	Yes/no	Yes*
Native TD	Native	Yes	No	No

*This includes cases in which folded subunits or complexes are ejected from a precursor complex, as has been reported in surface-induced dissociation experiments [15], as well as in CID of charge-reduced complexes [16]

## Proposed New and Updated Terminology for Top-Down Experiments

### Intact Mass Measurement/Native MS

The most basic experiment, this entails the measurement of the mass of an intact protein or complex under denaturing or native solution conditions, respectively, without any controlled dissociation being performed. While, therefore, this method is not truly part of the top-down “toolbox” (and referring to it as “top-down” should be avoided), it is an important first step in any TD experiment, as it can reveal protein integrity and complex stoichiometry (under native conditions) and provide valuable clues about proteoforms, e.g.*,* sequence variants and possible presence of (non-)covalent modifications.

Valuable information and improved desolvation can be obtained by subjecting a natively ionized protein or complex to limited (collisional) activation. If this is performed in conjunction with, and prior to, ion mobility measurements, this type of collision-induced unfolding (CIU) experiment can provide valuable insight into (changes in) the stability of various elements of protein structure in the gas phase [17]. Increasing gas-phase activation leads to backbone cleavage and/or (for a complex) monomer ejection, and these experimental strategies will be discussed in subsequent sections.

### Complex-Up

Going a step beyond determination of the mass of a protein complex, this type of experiment involves ionizing a noncovalent protein-protein complex from a native-like solution, after which it is partially disassembled in the gas phase in a controlled manner, without (significant) backbone cleavage in any of the subunits. Even then, this method can provide information on the complex architecture and stoichiometry, analogous to a middle-up experiment (vide infra) in solution but without involving an enzymatic/chemical digestion step. For instance, if collision-induced dissociation (CID) is used, a monomer is typically ejected carrying away a disproportionately large fraction of the total net charge of the complex, with minimal salt or water adduction, allowing accurate mass measurement of the individual subunits making up the complex. Additionally, the remaining, charge-stripped complex will be shifted to higher *m/z*, which has been exploited in the past for separation of overlapping signals from a polydisperse complex [18, 19]. Another important dissociation method used in complex-up MS is surface-induced dissociation (SID), which can result in ejection of subcomplexes that remain noncovalently bound, providing information on subunit connectivity in the original (larger) complex [15, 20, 21]. While complex-up work so far has primarily focused on the analysis of multi-protein complexes, we note that this can be extended to other types of complexes (protein-peptide, protein-RNA, etc.) and suggest that the same term is also appropriate to use in these cases.

### Denaturing Top-Down

In these experiments, the precursor protein is ionized from a denaturing solution, resulting in an extended gas phase conformation and high charge states of the (usually monomeric) protein. Dissociation is usually facile, as no significant noncovalent contacts need to be overcome to effect product ion release, although ECD/ETD with concurrent activation via collisions and/or infrared photons in a so-called activated-ion ECD/ETD experiment often improves dissociation efficiency and cleavage coverage [22–25]. The denaturing top-down (dTD) approach focuses on protein identification and sequence characterization by maximizing information on primary structure and is historically the first type of top-down experiment to have been demonstrated and reported [26–28]. Because of this, we propose that dTD should be the default interpretation of “top-down” (TD), if no more precise method is specified. A good recent example of the application of this type of experiment in the context of top-down proteomics is provided by the first pilot project of the CTDP in 2014, in which 74 proteoforms of histone H4 were identified using dTDMS [6]. “Deep sequencing” using two-dimensional MS with FTICR MS is a recently developed method that has shown promise to enhance cleavage coverage in dTDMS [29, 30]. Ionization and dissociation of intact proteins using MALDI in-source decay can be classified as a dTDMS experiment as well [31, 32].

### Complex-Down

This experiment aims to elucidate the stoichiometry of a noncovalent complex while simultaneously providing sequence information. Because of this, this approach combines the benefits of native MS with proteoform identification. Using native ESI and high-*m/z* transmission/detection, a given charge state of the gas-phase protein-protein complex is selected for dissociation. Subsequently, as in a complex-up experiment, vibrational activation ejects a relatively highly charged monomer, a process that is commonly assumed (although alternative mechanisms have been suggested [33]) to involve unfolding of the ejected monomer and (to a large extent) annihilation of higher order structure. The ejected monomer can in turn be subjected to what is effectively a dTD experiment by further activation. If this activation is performed after gas-phase isolation of the ejected monomer (i.e., in an MS^3^-type experiment), this is most easily carried out in an ion trap analyzer (with subsequent product ion detection using a high-resolution FTMS).

Alternatively, a pseudo-MS^3^ experiment can be performed by either subjecting all ejected products from the complex to dissociation (complicating data processing in case of non-identical subunits), or by performing monomer ejection by in-source (e.g.*,* nozzle-skimmer) activation and then isolating an ejected monomer for dissociation. The latter option has the downside of possibly creating ambiguity in assigning a highly charged monomer to a particular precursor complex, although during data processing, ions appearing only under harsh in-source conditions can be linked to CID products formed after isolating precursors that appear under gentle source conditions [34]. The term “complex-down” has been proposed by Wysocki and colleagues for experiments in which a complex is broken down into subunits and then further into covalent fragments [35]. The utility of this method for large protein complexes with monomer isolation has been demonstrated by Kelleher and colleagues [36, 37]. We propose that the term “complex-down” is appropriate whether or not gas-phase isolation of the ejected monomer is performed, and can therefore refer to either a one- or two-step process. It should be clear from the context how the term should be interpreted, and we feel it is not necessary at this point to propose separate terms for both. We again note that this methodology can be extended to other types of complexes, and these should also be referred to as complex-down experiments. The analogy between complex-up/complex -down and middle-up/ middle -down MS is obvious, and the analogous terms are distinguished by not involving an enzymatic digestion step in the former methods.

### Native Top-Down

Like complex-up and complex -down, this method relies on native (electrospray) ionization of monomeric proteins and noncovalent assemblies (although reports of ionization of intact peptide oligomers and protein complexes using MALDI have recently been published [38, 39]). In contrast to the aforementioned methods, the higher order structure is largely assumed to be retained during backbone cleavage in native top-down (nTD) experiments. Because of this requirement for selective backbone dissociation, advanced fragmentation methods such as ECD or ultraviolet photodissociation are typically used. In many cases, this leads to an observed dissociation pattern that is correlated to the secondary and tertiary structure of a protein or protein complex, and we propose that this type of experiment should be the default interpretation of “native top-down.” Both monomeric proteins as well as subunits within larger complexes can be probed with nTD, as illustrated in Figure 2. Examples can be traced back to the early 2000s, and cleavage sites have been linked to salt bridge patterns, presence of alpha-helices, crystallographic B factor, and surface exposure [19, 40–51]. The original “native ECD” experiment by Breuker and McLafferty also falls within the nTD category as, despite the fact that the cytochrome c dimer was disassembled in the gas phase, the observed fragmentation pattern revealed that backbone cleavage occurred while the protein was in a folded state [7]. In addition to probing the “native” state of the protein, probing of the gas-phase structure(s) after unfolding and refolding of the protein has also been performed [41, 52], as well as varying the level of gas-phase activation to generate a “melting curve” (i.e., plot of fragment intensity versus laser power or pre-activation energy) [53]. For the sake of simplicity, these studies will also be considered as nTD experiments in our framework, as they all lead to a fragmentation pattern that allows inference of the higher order structure. While both ionization and backbone cleavage in nTD are necessarily performed in a way that maintains higher order structure, in some studies ions were subsequently subjected to a limited (not sufficient to cause formation of *b* and *y* fragments) level of collisional activation to promote fragment release (somewhat similar to a complex-up experiment), under the assumption that structural information at that stage was already encoded in the sites of backbone cleavage [46, 47]. Radical-directed dissociation can be used as an alternative approach and primarily yields information on spatial proximity between cleavage sites and the site where the radical is generated [54].

Native top-down can also be used to determine the binding site(s) of noncovalent ligands, for example drugs, metal ions, and other small molecules such as spermine or 18-crown-6 [55–59] (identification of covalent binding sites is more efficiently performed using dTD methods [60, 61]). As is typical for nTD, a dissociation method is used that cleaves the protein backbone, while leaving noncovalent interactions intact, which leads to the masses of fragments that contain the binding site being shifted by the mass of the ligand. Experiments to map a protein/protein or peptide/protein interface with native top-down methods should also be grouped into this category [62, 63]. Conversely, tandem MS using an activation method that does disrupt noncovalent interactions and thus induces ligand release is also useful, as this can help identify the ligand. This type of experiment would fit into the complex-up category rather than native top-down.

While the above framework should prove useful for describing the majority of top-down studies described in the literature so far, there are no doubt cases where none of the terms defined here are fully accurate. For example, activated-ion ECD or ETD experiments are typically carried out using denatured proteins [22, 23]. If the protein were instead sprayed from native buffer, but with the higher order structure being mostly or completely annihilated in the gas phase prior to backbone cleavage, one could make the argument that this should no longer be considered a “native TD” experiment. Instead, this might merit the introduction of a new term, e.g., “activated-ion native TD.” Another type of ambiguity arises when considering very strongly bound ligands (e.g., certain instances of metal binding), where the (non)covalency of this interaction may be controversial. Such a case blurs the distinction between localization of a post-translational modification by denaturing TDMS and identification of the binding site of a noncovalent ligand by native TD. While it has long been established that CID of peptide-metal complexes often results in *b*/*y* fragments that carry the metal ion [64, 65], side-by-side comparisons with “softer” activation methods such as ECD have rarely been performed [56]. As it has therefore not been definitively established to what extent these observations inform on the native (solution) structure of these complexes, care should be taken when including this type of experiment in the nTD category, although this does seem to be the best fit among the classes presented here. If the need for more detail arises, authors may wish to modify one of the terms given in this paper.

Methods that provide information on protein primary structure (dTD, complex-down, nTD) are distinguished from those that do not (intact mass measurement/native MS, complex-up). We propose that the latter two should not be considered “true” top-down methods (see Figure 2). As explicitly implied by the term “top-down,” an intact, high-molecular weight protein (or complex) needs to be ionized, transmitted, and detected (i.e., getting to the *top*), and subsequently broken *down* to smaller fragments via gas-phase (backbone) dissociation (getting back “down” to a lower mass). In native MS (or intact mass measurement under denaturing conditions), the first step is accomplished, but no dissociation is performed. Similarly, as a protein complex is only separated into smaller subcomplexes or monomers (without cleavage of covalent bonds) in complex-up, it does not meet the proposed criteria listed above and is not considered among the top-down methods.

## Top or Middle? Up or Down?

The above discussion focuses primarily on the types of experiment made possible in recent years through improvements in MS technology. Considerable advances have also been made in sample preparation, leading to several experimental strategies worth mentioning (and defining unambiguously) in this context, particularly “middle-up” and “middle-down” (depicted at the bottom of Figure 1). Conceptually, these share some similarities with the complex-up/complex-down workflows described above, as (large) subunits are released from a larger assembly. The difference is that, in middle-up/middle-down, this release is performed in solution, by cleaving covalent bonds, rather than by disrupting noncovalent interactions in the gas phase. Currently, middle-up/middle -down studies have focused primarily on the analysis of monoclonal antibodies (mAbs), and the methods will be discussed with this application in mind (but methods for other types of proteins can also be accommodated).

### Middle-Up [66]

**Figure 3:**
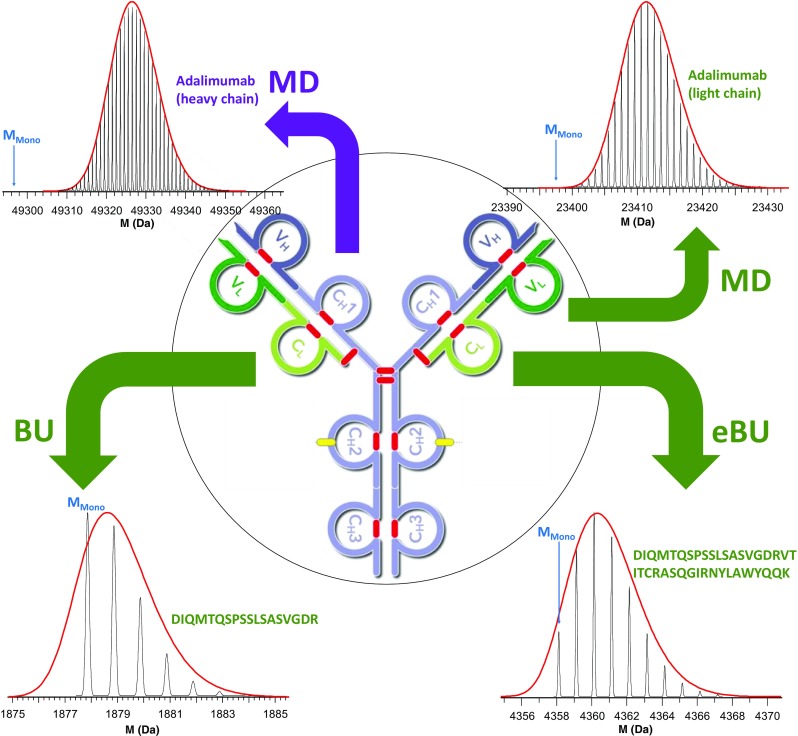
(Center) general structure of a monoclonal antibody (IgG1), surrounded by simulated (using Bruker DataAnalysis 4.1) isotope distributions for (top-left) the 49 kDa heavy chain, (top-right) the 23 kDa light chain, (bottom-left) an example 1.9 kDa enzymatically derived peptide consisting of the first 18 N-terminal light chain amino acid residues, and (bottom-right) an example 4.4 kDa enzymatically derived peptide consisting of the first 39 light chain N-terminal residues. The former two represent typical precursor masses for middle-up (MU) and middle-down (MD) MS, whereas the latter two fall within the bottom-up (BU) and extended bottom-up (eBU) mass range, respectively. Isotope distributions were simulated using the sequence of adalimumab, and the monoisotopic peak for each species (too low in intensity to be detectable for the heavy and light chain) is indicated in blue. The isotopic envelope is outlined using a solid red line, simulating the signal obtained at a resolution insufficient to resolve the individual isotope peaks

Effectively, this experiment entails intact mass measurement after cleaving the protein of interest into several large fragments/subunits via digestion. This can be achieved either chemically (e.g.*,* disulfide bond reduction or cyanogen bromide digestion to cleave C-terminal to methionine residues) [67], by stopping proteolytic digestion after a limited amount of time, or by using structure-specific enzymes that are selective for only one or a handful of cleavage sites in the protein. For example, for studies of mAbs, IdeS [68, 69], and GingisKHAN [70] allow cleavage in the lower and upper hinge region, respectively. Additionally, disulfide reduction can be employed to yield a limited number of relatively large fragments (either by itself or in combination with limited/restricted proteolysis, depending on the protein that is analyzed). An example of this is mAbs light and heavy chain analysis, as illustrated in Figure 3. Accurate (often isotopically resolved) mass determination of these large fragments then provides information on the protein integrity and possible modifications (but not the location of modifications or mutations), without the need for experimentally challenging accurate-mass determination of the intact, large protein. As depicted in Figure 3, however, the monoisotopic peak is usually too low in intensity to be observed, potentially complicating data analysis [71].

### Middle-Down

This term was first coined in 2009 [72], although the general concept predates this publication by a number of years [26, 73]. As in middle-up, digestion is used to cleave the protein of interest into large fragments/subunits, but rather than just a mass measurement of these fragments, gas-phase dissociation is subsequently performed [68]. This has the benefit of simplifying the experiment—specifically for mAb analysis; separating the heavy and light chain prior to MS/MS can facilitate spectral assignment, while disulfide reduction allows more complete unfolding and improves dissociation efficiency. As with top-down protein analysis, there exist large-scale applications of middle-up and middle-down protein analysis, referred to as middle-up and middle-down proteomics. As a side note, the first publication that proposed the “top-down” nomenclature also demonstrated the benefits of employing limited digestion to complement standard bottom-up data [26].

“Top-down” and “middle-down” are thus distinguished not based on precursor ion mass, but rather whether digestion (chemical or enzymatic) of a larger precursor occurred prior to MS analysis. However, we note that it is not always easy to draw the line between bottom-up and middle-down experiments, and “extended bottom-up” has been proposed as a term for peptides that fall in the intermediate mass range [74, 75]. We propose that rough guidelines can be defined based on the precursor mass. Following existing literature on this subject, we propose that “bottom-up” be the default term for precursors below 3 kDa, “extended bottom-up” for precursors between 3 and 7 kDa, and middle-down if the precursor mass exceeds 7 kDa [74]. In general, tandem MS studies become more challenging with increasing precursor mass, and we note that, while middle-down fragmentation of 25 kDa subunits (e.g., light chain or Fd subunit of a mAb) is feasible and sequence coverage of ca. 70% has been demonstrated [68], similar analysis of 50 kDa subunits (e.g., heavy chain or F(ab) subunit of a mAb) is still very challenging experimentally [76]. The increased precursor mass also has implications for data analysis, particularly as most data processing workflows rely on knowledge of the monoisotopic precursor mass. As shown in Figure 3, this becomes difficult or impossible to observe directly in many middle-down experiments. The different cases illustrated in Figure 3 use the mAb adalimumab (marketed as Humira for the treatment of arthritis) as an example, with simulated isotope distributions being displayed for the intact heavy and light chains, as well as a small and large peptides originating from in silico digestion of the light chain.

## Conclusion

Recently, both the number and sophistication of published top-down and middle-down protein analysis studies have been consistently increasing, with several multinational studies being either completed or underway. As the field becomes mainstream, and a large number of labs try their hand at even the most complex types of experiment using a variety of experimental methods and instruments, a certain degree of standardization of the field is now essential. As such, it is the authors’ hope that the proposed lexicon presented here will allow researchers to communicate about the various types of top-down and middle-down protein analysis in a clear, unambiguous way, paving the way for even more ambitious projects to be carried out in the future.
